# Use of seroprevalence to guide dengue vaccination plans for older adults in a dengue non-endemic country

**DOI:** 10.1371/journal.pntd.0009312

**Published:** 2021-04-01

**Authors:** Yi-Hua Pan, Mei-Ying Liao, Yu-Wen Chien, Tzong-Shiann Ho, Hui-Ying Ko, Chin-Rur Yang, Shu-Fen Chang, Chia-Yi Yu, Shu-Yu Lin, Pin-Wei Shih, Pei-Yun Shu, Day-Yu Chao, Chao-Ying Pan, Hong-Ming Chen, Guey-Chuen Perng, Chia-Chi Ku, Chwan-Chuen King

**Affiliations:** 1 Institute of Epidemiology and Preventive Medicine, College of Public Health, National Taiwan University (NTU), Taipei, Taiwan, Republic of China; 2 Department of Public Health, College of Medicine, National Cheng Kung University (NCKU), Tainan, Taiwan, Republic of China; 3 Department of Pediatrics, National Cheng-Kung University Hospital (NCKUH), College of Medicine, NCKU, Tainan, Taiwan, Republic of China; 4 Institute of Immunology, College of Medicine, NTU, Taipei, Taiwan, Republic of China; 5 Center for Diagnostics and Vaccine Development, Centers for Disease Control, Ministry of Health and Welfare, Taipei, Taiwan, Republic of China; 6 National Institute of Infectious Diseases and Vaccinology, National Health Research Institutes (NHRI), Tainan, Taiwan, Republic of China; 7 Institute of Microbiology and Public Health, College of Veterinary Medicine, National Chung Hsing University, Taichung, Taiwan, Republic of China; 8 Department of Health, Kaohsiung City Government, Kaohsiung, Taiwan, Republic of China; 9 Public Health Bureau, Tainan City Government, Tainan, Taiwan, Republic of China; 10 Institute of Basic Medical Sciences, College of Medicine, National Cheng Kung University (NCKU), Tainan, Taiwan, Republic of China; Duke-NUS GMS, SINGAPORE

## Abstract

A shift in dengue cases toward the adult population, accompanied by an increased risk of severe cases of dengue in the elderly, has created an important emerging issue in the past decade. To understand the level of past DENV infection among older adults after a large dengue outbreak occurred in southern Taiwan in 2015, we screened 1498 and 2603 serum samples from healthy residents aged ≥ 40 years in Kaohsiung City and Tainan City, respectively, to assess the seroprevalence of anti-DENV IgG in 2016. Seropositive samples were verified to exclude cross-reaction from Japanese encephalitis virus (JEV), using DENV/JEV-NS1 indirect IgG ELISA. We further identified viral serotypes and secondary DENV infections among positive samples in the two cities. The overall age-standardized seroprevalence of DENV-IgG among participants was 25.77% in Kaohsiung and 11.40% in Tainan, and the seroprevalence was significantly higher in older age groups of both cities. Although the percentages of secondary DENV infection in Kaohsiung and Tainan were very similar (43.09% and 44.76%, respectively), DENV-1 and DENV-2 spanned a wider age range in Kaohsiung, whereas DENV-2 was dominant in Tainan. As very few studies have obtained the serostatus of DENV infection in older adults and the elderly, this study highlights the need for further investigation into antibody status, as well as the safety and efficacy of dengue vaccination in these older populations.

## Author summary

The dengue virus (DENV) has rapidly spread out in tropical and sub-tropical regions. To measure the level of past DENV infections in the older population, we conducted a post-outbreak serosurvey in 2016 among a total of 4,101 older and elderly adults in Kaohsiung and Tainan cities in southern Taiwan. Our results showed district- and city-level differences in the overall seroprevalence of anti-DENV IgG antibody, increased seroprevalence with age, heterogeneous distributions of DENV serotypes, and similar percentages of secondary DENV infection in both metropolises, closely matching past histories of dengue outbreaks. This study demonstrates the importance of monitoring individual’s antibody status among the older and elderly population and highlights the need to investigate the safety and efficacy of dengue vaccination in these populations in the future.

## Introduction

Dengue, the most common arboviral disease, has expanded in recent years, with increasing numbers of outbreaks and a greater health impact worldwide, particularly in tropical and subtropical countries [1]. The causal agents are four distinct serotypes of dengue viruses (DENV), DENV-1 to DENV-4, and the virus is mainly transmitted by *Aedes aegypti* and *Aedes albopictus* [2–4]. The incidence of DENV infections has increased thirty-fold globally over the past fifty years [5]. Moreover, as the frequency of international travel has grown, dengue has become the leading cause of febrile illness among travellers. Travellers, at high risk of acquiring DENV infection in endemic areas, may play important roles in transmitting the virus to non-endemic regions [6–9]. That potential, alongside the significant geographic expansion of *Aedes* mosquitoes and DENV infection globally, as well as an aging population, has made an increase in dengue cases among older adults in dengue non-endemic countries and areas very likely [10]. Future public health needs in these populations must be investigated in advance [11,12].

Taiwan is located on the Tropic of Cancer in the western Pacific Ocean and has tropical and subtropical climates. Although several dengue outbreaks had been documented in Taiwan before World War II, including island-wide dengue outbreaks in 1931 and 1942–1943, no dengue cases were reported on the main island from 1945 to 1986 [13,14]. However, a large-scale outbreak of DENV-1 occurred on the main Island in 1987 following the lifting of both martial law and the ban on international travel [15–17]. Since then, imported dengue cases, especially from Southeast Asian countries, have played a significant role in triggering domestic outbreaks (S1 Table) [17–19]. In the past two decades, most local dengue outbreaks had occurred in southern Taiwan, with the greatest numbers of cases occurring in Kaohsiung City and Tainan City (S1 Fig) [20,21].

Dengue cases in Taiwan have predominantly occurred in adults, which is very different from the situation in Southeast Asia, where most cases occur in pediatric populations [14]. In addition, several studies following the 2002 dengue epidemic in Taiwan [22–24] found that older and elderly adults are at higher risk of developing severe dengue, previously known as dengue hemorrhagic fever/dengue shock syndrome (DHF/DSS). Investigators in Singapore, Malaysia and Vietnam also demonstrated an increased risk of high clinical severity and case fatality among the older population [25–27]. As the higher incidence of severe dengue and DHF/DSS cases has led to higher rates of hospitalization, admission to intensive care units (ICUs), and deaths in older adults, especially in the elderly, an effective dengue intervention strategy targeting older and elderly adults in non-endemic areas with DENV infection risk has become increasingly important.

Vaccination is considered as the most effective strategy of preventing infectious diseases. Currently, only a tetravalent dengue vaccine, Dengvaxia (CYD-TDV), has been licensed for dengue. However, this vaccine has been recommended solely for hyper-endemic areas for use in people who have had a previous DENV infection [28]. Due to the lack of information on DENV seroprevalence among older adults and the elderly in Taiwan, we measured DENV antibodies from healthy older adults and the elderly in two metropolises in southern Taiwan where most dengue outbreaks have occurred. As clinical severity of dengue varies by DENV serotypes as well as primary or secondary DENV infection [29,30], we also investigated the serostatus of each DENV serotype using a DENV NS1 serotype-specific IgG ELISA test, which can differentiate primary versus secondary DENV infection. This NS1 test has previously been used for serotyping of DENV and shows a high correlation with the traditional plaque reduction neutralization test (PRNT) in both clinical specimens and community-based epidemiological studies [31–33]. Other serotype-specific DENV-NS1 assays have also been used successfully in recent years [34,35]. Our retrospective cross-sectional seroepidemiological study with geographical variations provided a comprehensive view on the levels of DENV infection among the older population, which is important for evidence-based recommendations in vaccination and other public health programs in this frequently overlooked population.

## Methods

### Ethics statement

This study was reviewed and approved by the Research Ethics Committee of the National Health Research Institute (NHRI), Miaoli County, Taiwan (NHRI: EC1051108-R2). All data were anonymized; only the group data of age, gender, residential districts, and specimen-taken date were used in the data analysis in this study. No personally identifiable information was used as part of this study. As the 2015 dengue epidemic in Kaohsiung and Tainan involved the highest numbers of deaths in these two cities, local authorities at the Department of Health were responsible for taking blood samples from residents in the outbreak-affected areas in order to measure the magnitude of the DENV infection for future planning to prevent next outbreak, in accordance with the Communicable Disease Control Act—Article 43.

### Study design and sample collections

**Fig 1:**
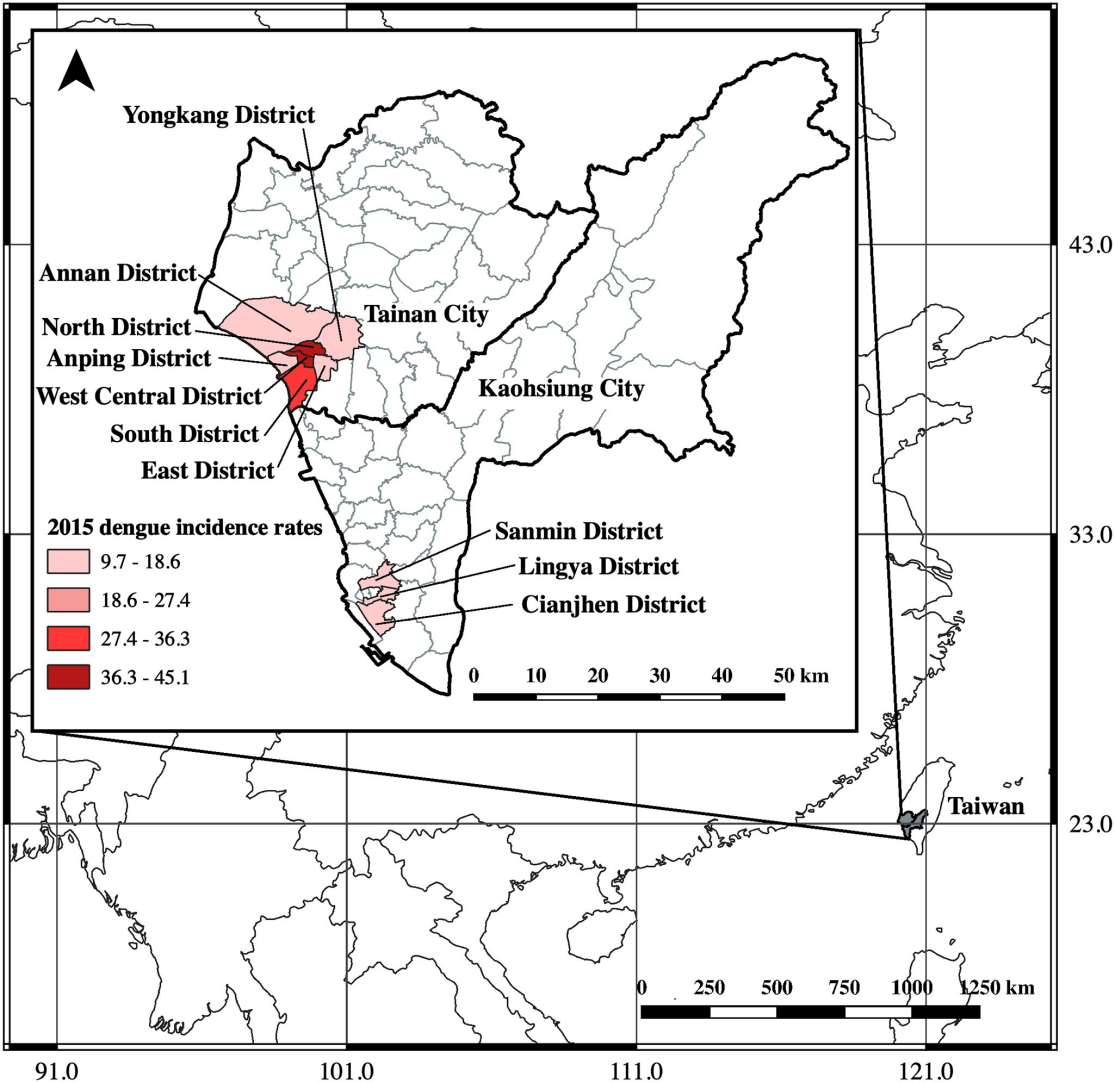
Location of Taiwan and map of study areas in Kaohsiung City and Tainan City. The areas shaded in grey color represent the study districts. The map was generated using QGIS software (QGIS Development Team, version 3.10, https://www.qgis.org.en/site/). The world (https://www.diva-gis.org/Data) besemap was obtained from DIVA-GIS, while basemaps of the Taiwan cites (https://data.gov.tw/dataset/7442) and Taiwan districts (https://data.gov.tw/dataset/7441) were all collected from the government’s open data platform.

Kaohsiung City and neighboring Tainan City in Taiwan experienced a severe dengue epidemic in 2015 with 19,723 and 22,760 indigenous laboratory-confirmed dengue cases, respectively [36,37]. During this epidemic, the population above 70 years of age had the highest dengue incidence rate, followed by those aged 60–69 and 50–59 years [21,37]. Therefore, we retrospectively collected serum samples from residents aged ≥ 40 years old living in Kaohsiung and Tainan who participated in an annual free health examination campaign for routine health screenings from January to June 2016. The aim of this annual health examination is to provide preventative healthcare against adult diseases and promote health in the older population. People aged 40–64 years can enroll in this free service once every 3 years, while those ≥65 years old (i.e., the elderly), and those over 35 years old with post-polio syndrome can enroll annually. The detailed health examination includes blood biochemistry tests, a renal function test and a urine test. Kaohsiung and Tainan residents who meet the enrollment criteria can participate in the health examination campaign regardless of their health status. In this study, we collected 1498 and 2603 serum samples in total from the three districts in Kaohsiung that had had the most frequent past dengue outbreaks and from seven districts in Tainan, respectively. All of the selected districts had over 500 dengue cases in the 2015 outbreak, and the district-specific incidence of dengue ranged from 9.7 to 45.1 per 1,000 population (Fig 1). The percentages of elderly study subjects (≥ 65 years) were 84.45% (1265/1498) in Kaohsiung and 40.68% (1059/2063) in Tainan. Detailed information for each district is shown in S2 Table [38,39].

### Serological tests

**Fig 2:**
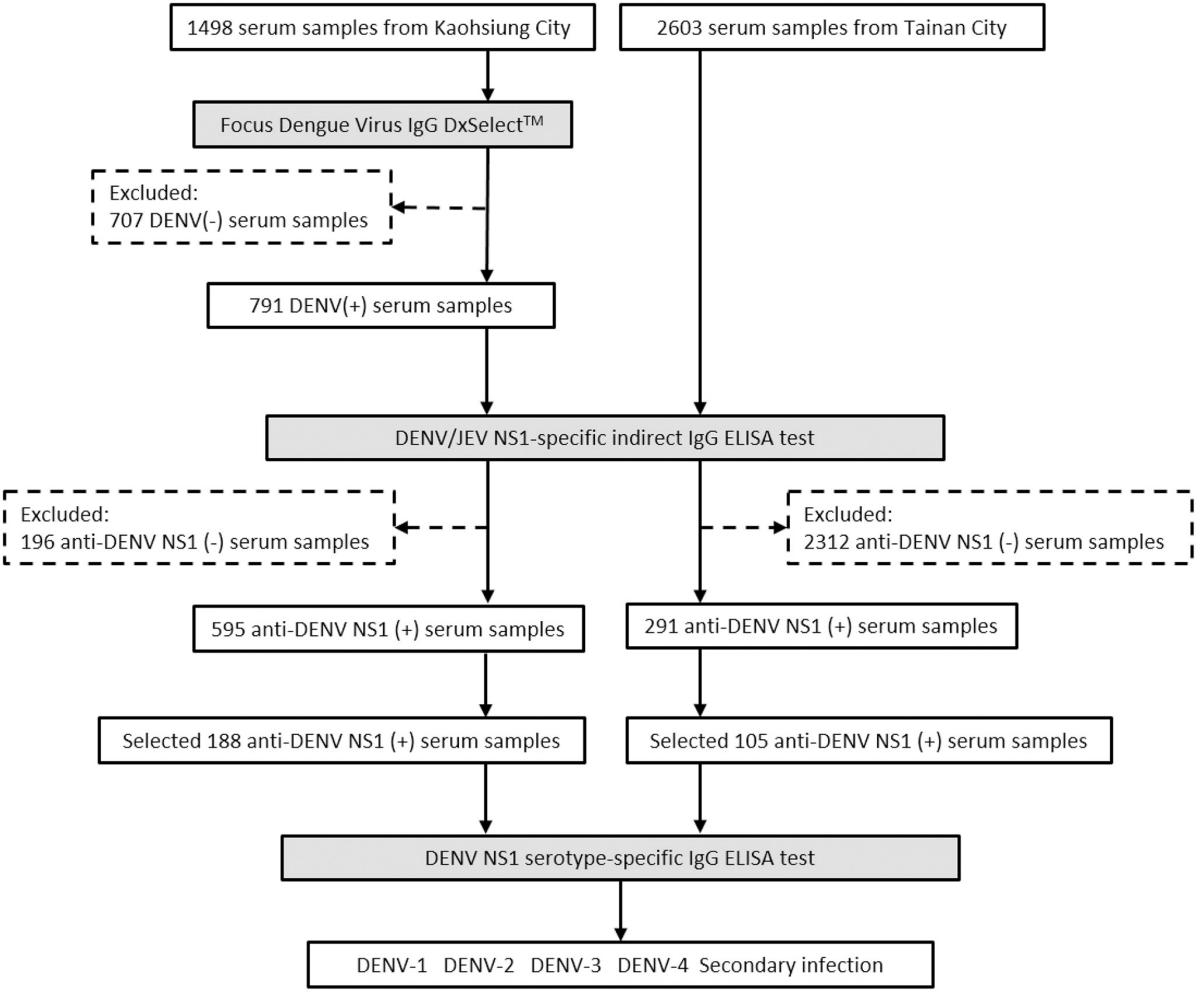
Flow chart of serosurvey of DENV-IgG among study subjects in Tainan City and Kaohsiung City.

Three serological tests were included in this study. First, Focus Dengue Virus IgG DxSelect indirect enzyme-linked immunosorbent assay (ELISA) with a sensitivity of 96% and specificity of 93% [40] was used to test 1498 serum samples from Kaohsiung City for DENV-specific IgG antibodies. Second, DENV/JEV NS1-specific indirect IgG ELISA was also employed. This test can differentiate between DENV and JEV in accordance with following the protocols as listed in the previous study [41] and also used monoclonal antibody (mAb) D2/8-1 (YH0023, Yao-Hong Biotechnology Inc.), as the NS1-capture antibody for testing the anti-DENV-IgG-positive samples from Kaohsiung and all the 2603 samples collected directly from Tainan. This mAb D2/8-1 can recognize a conserved linear epitope of NS1 protein among the four DENV serotypes and JEV [42]. In brief, the mixture of DENV-1, 2, 3, 4 infected Vero cell supernatants containing soluble DENV-NS1 in one well, the JEV-infected Vero cells in another well, and the cell control in the 3^rd^ well, were all added into the mAb D2/8-1 coated plates. Finally, the human serum samples were added to measure DENV or JEV antibody. The DENV-tested results were also compared with the JEV-tested results to get a comprehensive serological profile. “DENV-seropositive” was defined as having a ratio ≥ 2.0 of the tested optical density (O.D.) reading divided by the O.D. value of negative cell control for each sample, whereas “DENV-seronegative” was defined as having such a ratio < 2.0 [31]. Third, DENV-NS1 serotype-specific IgG ELISA test (Fig 2) was applied to 188 and 105 serum samples with positive DENV-NS1-IgG considering variations in age groups and residential districts from Kaohsiung and Tainan, respectively, to measure both DENV serotyping and secondary DENV infection simultaneously, based on the protocols of the Taiwan-CDC [31]. This simple, sensitive, and specific assay can identify the DENV serotypes of primary infection using convalescent-phase or post-infection serum samples [31,32]. The performance of this assay has been shown to be correlated closely with dengue virus PRNT [89.7% (73+32/117) consistency of primary and secondary DENV infection [31–33], and the correlation of Kappa statistics = 0.776] [33]. Examples of the data from this DENV NS1 serotype-specific IgG ELISA test for DENV-serotyping are provided (S4 Table) to elucidate the calculations for determining DENV serotype and secondary infection. Each well contained the supernatant of only one single serotype of DENV-1, 2, 3, 4-infected Vero cells in the four separate individual wells. All these viruses were quantitated by plaque assays and adjusted to have the same amount of the virus titer to interact with the coated mAb D2/8-1. In this way, the primary and secondary DENV infection could be differentiated very clearly. “DENV serotypes” were finalized from only those samples with primary DENV infection. In data analyses, the DENV serotype was determined by the ratio of the of the OD values for the two highest DENV serotypes with OD ratios of ≥ 1.2, where the higher OD value was determined to be the infecting DENV serotype. “Secondary DENV infection” was defined by at least two of the four OD values from the four DENV serotypes having similar values (OD ratios <1.2) and a ratio of ≥2.0 for the tested serotype OD values divided by the individual negative control [31–33]. The detailed laboratory procedures are described in S1 Protocol.

In addition, the seroprevalence of DENV-IgG from 2603 Tainan serum samples was measured directly using 2^nd^ and 3^rd^ assays in Fig 2. The solid examples of data analyses are described in the S3 Table.

### Data analyses and statistical tests

Age-standardized seroprevalence of anti-DENV IgG was calculated for each city using the combined age-structured population in these two cities in 2016 as a reference population. The detailed calculations of this age-standardized seroprevalence of anti-DENV IgG are described in the S5 Table.

**Fig 3:**
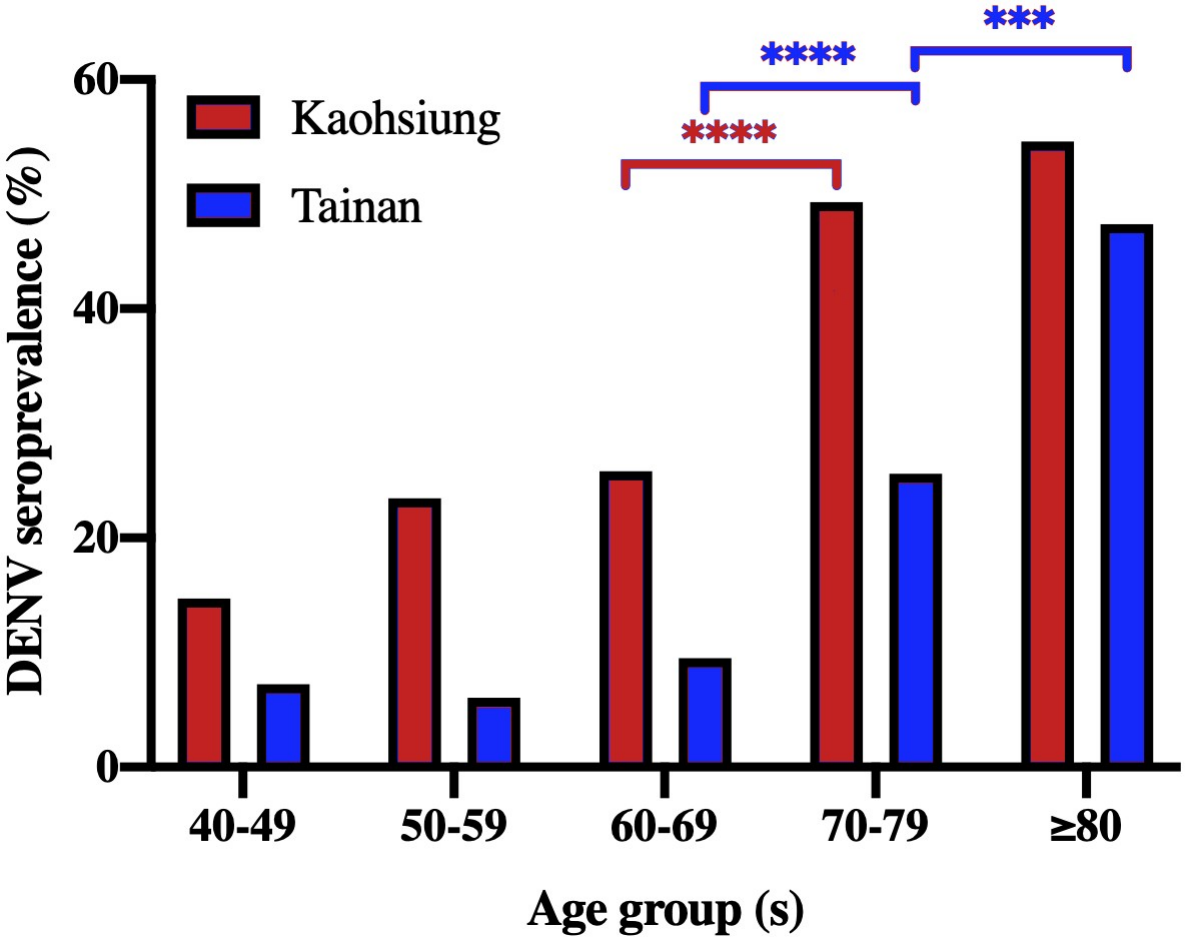
The seroprevalence of DENV-IgG distributions of four DENV serotypes by age among study subjects in (A) Kaohsiung and (B) Tainan cities of southern Taiwan, 2016.

Chi-square tests were performed to examine the difference in DENV seroprevalence between each age groups in Fig 3. Chi-square test for trend was used to demonstrate the patterns of increasing DENV seroprevalence with age (Table 1).

**Table 1:** Age-, gender-, and district-specific seroprevalence of anti-DENV IgG in Kaohsiung City and Tainan City in 2016.

No of Study Subjects	1498 subjects in Kaohsiung			2603 subjects in Tainan		
Demographic Characteristics	No. positive/No. tested	Seroprevalence (%)	p-value	No. positive/No. tested	Seroprevalence (%)	p-value
Gender						
Male	309/799	38.67		132/1302	10.14	
Female	286/699	40.92		159/1301	12.22	
Age Groups (years)						
40–49	5/34	14.71	**<0.0001**^b^	28/387	7.24	**<0.0001**^b^
50–59	23/98	23.47		47/776	6.06	
60–69	130/503	25.85		95/1000	9.50	
70–79	318/645	49.30		103/402	25.62	
≥80	119/218	54.59		18/38	47.37	
Total	595/1498	39.72		291/2603	11.18	
Age-standardized^a^		**25.77**			**11.40**	

^a^ Age-standardized seroprevalence of anti-DENV IgG used the age structure of the total population in Kaohsiung City and Tainan City in 2016 as the reference population (see detailed calculations in the S5 Table).

^b^ Chi-square for Trend by age group

Univariable and multivariable logistic regression analyses were used to compare categorical variables, including age, gender, and residential districts, using SAS 9.4, and *p* < 0.05 was considered statistically significant. The geographic figure was presented using Quantum geographic information system (QGIS Geographic Information System, version 3.10) [43] and GraphPad Prism 8.0 (GraphPad Software, Inc., San Diego, CA).

## Results

### General characteristics of study subjects in Kaohsiung and Tainan

Table 1 shows the general characteristics of 1498 study subjects (ranging 40–96 years) in Kaohsiung City and 2603 participants (ranging 40–88 years) in Tainan City. The mean age of study subjects in Kaohsiung was significantly older than that in Tainan (70.94 ± 8.70 vs. 60.13 ± 9.97, p < 0.0001, two-tailed independent t-test). However, the numbers of male and female participants were almost equally distributed in both cities.

### Seroprevalence of anti-DENV IgG in Kaohsiung and Tainan

#### Overall seroprevalence

The overall seroprevalence of anti-DENV IgG among participants in Kaohsiung was significantly higher than that in Tainan (39.72% and 11.18%, p < 0.0001). Using the combined standard population in the two cities in 2016, the age-standardized seroprevalences were 25.78% and 11.40% in Kaohsiung and Tainan, respectively (Table 1).

#### Age- and gender-specific seroprevalence

The age-specific seroprevalences of anti-DENV IgG significantly increased with elevated age in both cities (p<0.0001), and DENV seroprevalence results in all age groups in Kaohsiung were higher than those in Tainan (Table 1). Interestingly, sharp increases in DENV seroprevalence from 60–69 years to 70–79 years of age were observed in both cities (Fig 3).

With regard to gender-specific seroprevalence, females showed a slightly higher seroprevalence of anti-DENV IgG than males in both cities (Table 1), but the difference did not reach statistical significance (Kaohsiung: *p* = 0.3763; Tainan: *p* = 0.0921) (Table 2).

**Table 2:** Univariate and multivariable analyses of past DENV infection among study subjects in in Kaohsiung City and Tainan City in 2016.

City/ Characteristics	No. Sero(+)/No. tested	Seroprevalence (%)	Univariate analysis			Multivariable analysis		
			Crude OR	95%CI	p-value	Adjusted OR	95%CI	p-value
**Kaohsiung**								
**Gender**								
Male	309/799	38.67	1	ref.	-	1	ref.	-
Female	286/699	40.92	1.098	0.892–1.351	0.3763	1.357	1.088–1.692	0.0068
**Age Groups (years)**								
40–49	5/34	14.71	1	ref.	-	1	ref.	-
50–59	23/98	23.47	1.778	0.617–5.121	0.2862	1.737	0.601–5.019	0.3079
60–69	130/503	25.84	2.021	0.766–5.330	0.155	2.109	0.796–5.588	0.1334
70–79	318/645	49.30	5.639	2.156–14.749	0.0004	6.255	2.372–16.494	0.0002
≥80	119/218	54.59	6.97	2.601–18.678	0.0001	8.052	2.970–21.831	<0.0001
**Districts**								
Sanmin	207/547	37.84	1	ref.	-	1	ref.	-
Cianjhen	231/571	40.46	1.116	0.877–1.419	0.3711	1.014	0.788–1.305	0.9131
Lingya	157/380	41.32	1.156	0.885–1.511	0.287	0.831	0.625–1.105	0.2024
**Tainan**								
**Gender**								
Male	132/1302	10.14	1	ref.	-	1	ref.	-
Female	159/1301	12.22	1.234	0.966–1.576	0.0921	1.26	0.975–1.628	0.0773
**Age Groups (years)**								
40–49	28/387	7.24	1	ref.	-	1	ref.	-
50–59	47/776	6.06	0.827	0.509–1.342	0.4413	1.019	0.618–1.681	0.9415
60–69	95/1000	9.50	1.346	0.868–2.087	0.1846	1.387	0.882–2.181	0.1563
70–79	103/402	25.62	4.417	2.830–6.892	<0.0001	4.663	2.958–7.350	<0.0001
≥80	18/38	47.37	11.54	5.485–24.282	<0.0001	12.837	5.984–27.541	<0.0001
**Districts**								
Annan	17/466	3.65	1	ref.	-	1	ref.	-
Anping	8/158	5.06	1.409	0.596–3.330	0.4351	1.519	0.636–3.626	0.3466
East	30/398	7.54	2.153	1.169–3.966	0.0138	1.89	1.015–3.519	0.0448
Yongkang	102/760	13.42	4.094	2.416–6.937	<0.0001	2.937	1.707–5.501	<0.0001
South	54/400	13.50	4.122	2.348–7.237	<0.0001	3.793	2.140–6.721	<0.0001
North	29/184	15.76	4.942	2.643–9.240	<0.0001	3.861	2.033–7.333	<0.0001
West Central	51/237	21.52	7.242	4.076–12.868	<0.0001	7.109	3.949–12.796	<0.0001

**OR**: Odds Ratio; **CI**: Confidence Interval.

### Univariable and multivariable analyses of factors associated with DENV-IgG seroprevalence

In univariate logistic regression, residents aged 70–79 and ≥80 years showed significantly higher seroprevalence of DENV-IgG than those aged 40–49 years serving as the reference groups in the two cities (p<0.0001) (Table 2). Residents living in five of the seven residential districts in Tainan City additionally had significantly higher DENV seroprevalences (p<0.05).

Multivariable logistic regression demonstrated that age ≥70 years and being female were the two independent risk factors for past DENV infection in Kaohsiung, whereas age ≥70 years and residential district were risk factors in Tainan (Table 2).

### DENV serotypes and secondary DENV infection

#### DENV serotypes in Kaohsiung and Tainan

**Fig 4:**
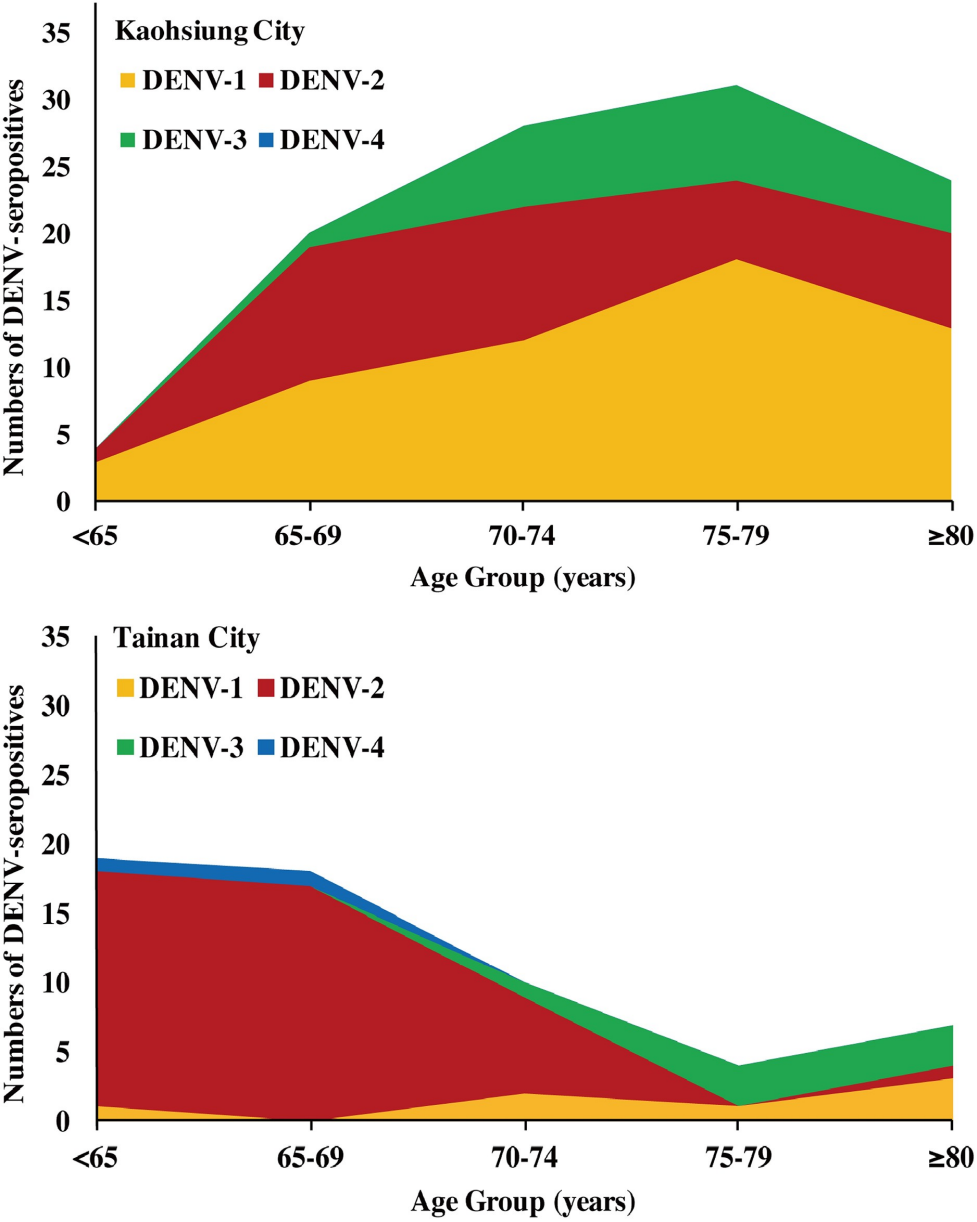
The distributions of four DENV serotypes among different age groups of study subjects with primary DENV infection in (A) Kaohsiung and (B) Tainan cities of southern Taiwan, 2016.

The serotyping results of 188 and 105 selected individuals from Kaohsiung and Tainan showed different distributions of DENV serotypes (Fig 4). In Kaohsiung, DENV-1 ranked highest [29.26% (55/188)], followed by DENV-2 [18.09% (34/188)], and DENV-3 [9.57% (18/188)]. DENV-1, and DENV-2 covered wider age groups and DENV-3 appeared primarily in residents ≥70 years old. However, there was no DENV-4 seropositive individuals in Kaohsiung City. In contrast, DENV-2 ranked highest [40% (42/105)] in Tainan City, followed by DENV-1 [6.67% (7/105)], DENV-3 [6.67% (7/105)], and DENV-4 [1.90% (2/105)].

*Secondary DENV infection*. The percentages of secondary DENV infection in residents of Kaohsiung and Tainan were very similar, without significant difference [43.09% (81/188) and 44.76% (47/105), respectively, *p* = 0.7814] (S6 Table).

## Discussion

We conducted a retrospective dengue seroepidemiological survey using serum samples from healthy residents ≥ 40 years of age in districts from two metropolises in southern Taiwan and observed both district- and city-level differences in DENV seroprevalence. The DENV seroprevalence increased with age, and there was a sharp rise from the age groups of 60–69 to 70–79 years in both Kaohsiung and Tainan cities. Our study also confirmed that past dengue epidemics in these two cities were mainly caused by DENV-1 and DENV-2.

Seroprevalence of DENV infection reflects not only the magnitude of total infection, but also helps identify high-risk areas. The age-standardized seroprevalence of anti-DENV IgG in Kaohsiung was two times higher than that in Tainan (25.77% vs. 11.40%, respectively), which is consistent with the observation that Kaohsiung had had more frequent and larger dengue outbreaks during past dengue epidemics than Tainan did (S1 Table and S1 Fig) [37]. These results provide evidence of both district- and city-level differences in past DENV infection in southern Taiwan, and could help public health leaders plan future vector control programs, such as releasing *Wolbachia*-infected mosquitoes [44] and health education programs for both clinicians and local residents [45] while considering geographical variations and differences in age-specific seroprevalence results in dengue affected areas.

The age distribution of dengue cases is one of the differences in epidemiological characteristics between non-endemic and endemic countries [46,47]. Dengue in Taiwan has not reached endemic status [13,48,49], and our results show the differences in affected-age groups and a lower seroprevalence in southern Taiwan compared to those in dengue-endemic regions. However, DENV seroprevalence consistently increased with age [50,51], much as has been observed in dengue endemic regions [52]. The age-specific DENV seroprevalence results also verify previous local epidemics. The sharp increase in age-specific seroprevalence from the 60–69 to 70–79 year-old in southern Taiwan is consistent with the past findings [16,51] and also confirms the large-scale epidemic in 1942–1943 [17]. These results support that the most dominant populations pre-exposed to DENV infection in southern Taiwan are older adults and the elderly [36], which is quite different from the striking increases among younger age groups (<35 years old) in South American and Southeast Asian countries [53–55].

Several studies have shown that the elderly is more likely to develop DHF, DSS, and severe dengue than the younger population in Taiwan [23,24]. Three major reasons may contribute to the severity of dengue illness in the elderly: immunosenescence with decreases in innate and adaptive immune functions (i.e. reduced humoral and cell-mediated immune responses) [56–58], greater prevalence of comorbidities and co-infection [36,59], and higher probability of acquiring secondary DENV infection [10,30]. In our study, the percentage of pre-exposure to the infection of two or more DENV serotypes in both cities were close to 40%, which indicates that the percentages of people with primary DENV infection might be more than 50% of the DENV seropositive subjects in the study. These people who had been infected once might be at risk for more severe dengue resulting from future secondary heterologous DENV infection, which increases the likelihood of severe dengue illness [60]. All these findings highlight the importance of active surveillance of the DENV among Taiwan’s elderly, in whom the illness is more likely to progress to severe dengue. Vaccination of previously DENV infected older populations should be recommended to prevent severe disease. However, the currently available DENV vaccine, Dengvaxia, based on live-attenuated yellow fever 17D-dengue chimeric vaccine, would be contradicted in DENV-naïve older individuals as it could increase the risk of hospitalization and severe disease [61]. Therefore, a DENV serology test should be conducted first to ensure only DENV-positive individuals receive this vaccine [62]. Unfortunately, the efficacy of Dengvaxia is much lower against DENV-1 and DENV-2 (34.7%-54.8%) than against DENV-3, and DENV-4 [63]. This indicates that Dengvaxia might not be suitable for rollout in Taiwan. As several dengue vaccines are in the later phase of human clinical trials, future dengue vaccines need to provide a broader immune responses to all DENV serotypes in both naïve and previously exposed individuals.

Large-scale seroepidemiological studies need convenient serological tests. For DENV, the envelope (E) protein is the primary target of neutralizing antibodies [29,64]. Although PRNT has been considered the “gold standard” for confirmation of antibodies in dengue vaccine evaluation [65] and serotyping of past DENV infection [66–71], the test is time-consuming and laborious. More importantly, there is high cross-reactivity of viral envelope (E)- and membrane (M)-specific immunoglobulin G (IgG) antibodies elicited from natural infection or vaccination by other flaviviruses; JEVs in particular are still active in Taiwan [72], and a JE vaccine immunization program has been extended to adults during JE outbreak seasons in Kaohsiung City in recent years [73]. We chose a DENV NS1 serotype-specific IgG ELISA, which is more appropriate for testing large numbers of serum samples. The NS1 IgG ELISA can differentiate infection serotypes among subjects pre-exposed to a single DENV infection, as its serotyping results for DENV from individuals with primary DENV infection correlates well with the outbreaks caused by DENV-1, 2, 3, and 4 in different years [16]. Similar findings on Liuchiu Township in southern Taiwan have confirmed the usefulness of this assay in field seroepidemiological studies [33]. Since Taiwan mostly has primary DENV infections [74,75], we were able to obtain substantial DENV serotyping results in the study. We found that 42.69% (540/1265) and 16.71% (177/1059) of study subjects ≥ age 65 years in Kaohsiung and Tainan, respectively, were susceptible to the non-experienced DENV infection, DENV-4, which was the lowest experienced serotype across residents of both cities. This result is consistent with epidemiological findings that DENV-4 was associated primarily with sporadic cases and small clusters without a large-scale epidemic in Taiwan (S1 Table). DENV-2 was dominant in Tainan, implying that Tainan residents were more likely to be susceptible to multiple other DENV serotypes, and the serotype-specific susceptibility indicates that potential future risk will be present at local levels. Our results also imply a need to monitor DENV serotypes in local areas serving for sentinel surveillance through enhanced integrated dengue surveillance (i.e., integrated clinical, virological, serological and mosquito surveillance).”

This study has several limitations. First, we only recruited healthy older adults and the elderly mostly in dengue high incidence districts among our study subjects, which might result in selection bias. Second, the serological test that detects IgG against flaviviral NS1 antigen might underestimate DENV seroprevalence, as anti-NS1 antibodies have been regarded as short-lived, in particular IgG3, compared to anti-E antibody, which remains present for decades in human serum samples post-infection. Third, the serotype-specificity of DENV NS1 IgG ELISA ranged from 75%-80% in primary DENV infection, which is lower than that of PRNT and requires further verification [33]. Fourth, the cross-reactivity of antibodies between DENV and Zika virus was not determined in this study because there were only three imported Zika cases, with no indigenous cases during the study period [76] and only a very low seroprevalence of Zika virus infection [ranging from 0.47% (1/212) to 4.2% (9/212)] was documented in Tainan [77]. Finally, the lack of individual data on comorbidities and past history of dengue illness, as well as other environmental factors of the living areas limit our understanding of the risk factors for acquiring DENV infection and/or severe dengue illness among older adults and the elderly. Nonetheless, the important findings regarding a higher seroprevalence of DENV infection in the elderly, and geographical variations between the two cities can prepare decision-makers to conduct better public health plans to prevent threats of dengue in future years.

Human population growth and the rapid expansion of *Aedes* mosquitoes [78] coupled with increased international travel [79] and an aging population, may endanger the susceptible elderly population in the areas of dengue-non-endemic countries with limited health resources at higher risk for severe dengue and fatality [80–82]. Dengue vaccination might be a possible solution for minimizing the risk of developing severe dengue in such a high-risk population, if a safe and effective vaccine is available. To the best of our knowledge, this is the first study that provides the information on DENV serostatus among the older population. Our results highlight the importance of serological testing for DENV before considering vaccination [62,81], as well as targeting high-risk areas and high-risk population for enhanced dengue surveillance, vector control, and public health education programs on preventive measures [83]. Secreted soluble NS1 (sNS1) is easily detected in acute serum samples of dengue patients [34,84–86] and the conserved region of NS1 among DENV-1, 2, and 3 serotypes is located in the β-ladder domain (S3 Fig), while different DENV serotypes vary in different domains of the NS1 protein (S2 Fig), using the methods we described previously [87]. The immune-epitope results also showed DENV serotype-specific amino acid residues (S4 Fig and S1 Dataset in Supporting Information) [88]. As older age groups are susceptible to and at risk for severe dengue, future efforts should improve the specificity of DENV-NS1 serotyping. This test needs to minimize cross-reactivity among different DENV serotypes and across various flaviviruses, in order to help seroepidemiological studies and serodiagnosis of DENV serotypes, as well as facilitate an understanding of the pathogenesis mechanism of severe dengue more efficiently [29,84,85, 89–91]. To date, the safety and efficacy of dengue vaccines in older adults and the elderly remain unknown, as most clinical trials have not included this population [92]. It is critically important to investigate the safety and efficacy of dengue vaccines in this older population under different epidemiological conditions.

In summary, active DENV serosurveillance in regions of Southern Taiwan that experience periodic DENV outbreaks is critical for the development of evidence-based control measures against dengue. Serosurveillance and mosquito surveillance to monitor the population of *Aedes* indoors and outdoors will provide information to guide the best prevention and control programs for dengue, including vaccination, vector control, and health education to reduce mosquito breeding sites, prevent mosquito bites, and mobilize communities undergoing routine evaluations. Taken together, these are key measures that can be taken to minimize the threat of dengue to the elderly.

## Supporting information

S1 Table Years, areas, numbers of confirmed dengue cases, DENV serotypes, and possible sources of major large-scale dengue epidemics in Taiwan, between 1915 and 2015. (PDF)

S2 Table Population sizes, population densities, dengue cases and district-specific incidence of dengue in Kaohsiung City and Tainan City, 2015. (PDF)

S3 Table Final results of DENV and/or JEV infection for three samples, based on the ratio of OD values. (PDF)

S4 Table Five tested samples and their final results of DENV serotype or Secondary DENV infection judged, based on the OD value ratio. (PDF)

S5 Table Calculation process and steps to obtain the age-standardized DENV-IgG seroprevalence in Kaohsiung City and Tainan City, 2016. (PDF)

S6 Table Geographical variations in DENV serotypes and secondary DENV infection in the five age groups in Kaohsiung and Tainan cities of southern Taiwan, 2016. (PDF)

S1 Fig Annual numbers of indigenous and imported laboratory-confirmed dengue cases in (A) Kaohsiung City and (B) Tainan City from 1998 to 2015. (TIF)

S2 Fig Amino acid variations of NS1 among the four DENV serotypes and JEV. In total, 9,762 DENV strains and 308 JEV strains collected worldwide from the ViPR database (during 1950–2020) [88] with known DENV serotype and available NS1 sequences were analyzed. These included 4,085 DENV-1 strains (12 Taiwan strains), 3,259 DENV-2 strains (107 Taiwan strains), 1,776 DENV-3 strains (17 Taiwan strains), 642 DENV-4 strains (2 Taiwan strains), and 308 JEV strains (30 Taiwan strains). The three domains of NS1 are: (1) β-roll domain [amino acid (a.a.) residues 1–30], (2) Wing domain [a.a. residues 37–152], and (3) β-ladder domain [a.a. residues 180–352]. The plot shows that the Wing domain of DENV NS1 protein had the highest a.a. variation. Among the four DENV serotypes, DENV-4 had the highest a.a. variation percentages in all the three domains of the NS1 protein. In addition, both DENV-1 and DENV-2 had the highest a.a. variations in the Wing domain, whereas DENV-3 showed the greatest a.a. variation in the β-ladder domain; and DENV-4 revealed the widest a.a. variation in the β-roll domain. The values of mean ± standard deviation (mean ± SD) are shown for each of the three domains for all of four of the DENV serotypes. The methods used to calculate the percentage of amino acid variations were described previously [87].
(TIF)

S3 Fig Specific signatures of among the four DENV serotypes and JEV. The DENV serotype- and JEV-specific consensus amino acid signatures for each group are shown by the constellation of the 352 amino acid residues in NS1 protein (distinguished by darkened colors). The plot shows that the Wing domain had the highest signatures to differentiate the DENV serotypes from JEV. Conversely, β-ladder domain sequence was more conserved among the four DENV serotypes. The methods used to find signatures were described previously [87].
(TIF)

S4 Fig DENV-1-, DENV-2-, and DENV-3-specific amino acid signatures in the NS1 protein and evidence-based immune-epitopes. The DENV-1-, DENV-2-(Fig. S4A), and DENV-3 (Fig S4B)-specific consensus amino acid signatures for each DENV serotype are shown by the constellation of the 26 evidence-based immune-epitopes documented in the literature, which are listed in the letters within the boxes. All evidence supporting information from the raw experimental data for these immunological epitopes is listed in Supporting Information file from ViPR databases [88].
(TIF)

S1 Protocol Laboratory procedures of the three serological assays used in this study. (PDF)

S1 Dataset Immune-epitope dataset. (XLSX)

S1 STROBE Checklist (PDF)
