# Supplementary material for: Use of seroprevalence to guide dengue vaccination plans for older adults in a dengue non-endemic country
Source: PLoS Negl Trop Dis. 2021 Apr 1;15(4):e0009312. doi: 10.1371/journal.pntd.0009312 (PMC8075253; doi:10.1371/journal.pntd.0009312)
Supplement: S1 Table — (PDF) [file pntd.0009312.s001.pdf]

S1 Table. Years, areas, numbers of confirmed dengue cases, DENV serotypes, and possible sources of major large-scale dengue epidemics in Taiwan, between 1915 and 2015.

Year(s) [c]	Taiwan		DENV Serotype identified	Possible source of the DENVs from outbreaks[a]	Major Epidemic Areas
	Indigenous cases	Imported cases			
**1915-1916**	Island-wide dengue outbreaks documented without the information of dengue cases				
**1931**					
**1942-1943**					
**1981**	>8000	-	DENV-2	Philippines	Isolated islet of Hsiao-Liu-Chiu in Pingtung County[d]
**1987**	>527	-	DENV-1<br>DENV-2	Thailand<br>Philippines	Pingtung County & Kaohsiung City<br>Kaohsiung City
**1988**	>4389	-	DENV-1	Thailand	Southern Taiwan[b]
**1989**	16	19	-	-	
**1990**	0	10	-	-	-
**1991**	149	26	DENV-1	Thailand	
**1992**	4	19	-	-	-
**1993**	0	13	-	-	-
**1994**	222	22	DENV-3<br>DENV-1	Philippines<br>Vietnam	Kaohsiung City<br>Tainan City
**1995**	329	40	DENV-1<br>DENV-1<br>DENV-3	Vietnam<br>Malaysia<br>Philippines	New Taipei City<br>Pingtung County<br>Kaohsiung City
**1996**	20	35	DENV-1	Thailand	Taipei City
**1997**	19	57	DENV-2	Indonesia	Tainan City
**1998**	238	110	DENV-3<br>DENV-2<br>DENV-2	Thailand<br>Thailand<br>Indonesia	Tainan City<br>Kaohsiung City<br>Kaohsiung City
**1999**	42	26	DENV-1	Vietnam	Kaohsiung City
**2000**	113	26	DENV-4	Thailand	Tainan City
**2001**	227	54	DENV-2	Philippines	Kaohsiung City
**2002**	**5,336**	52	DENV-2<br>DENV-1	Philippines<br>Indonesia	Southern Taiwan[b]<br>Pingtung County & Kaohsiung City
**2003**	86	59	DENV-2	Philippines	Pingtung County & Kaohsiung City
**2004**	336	91	DENV-1<br>DENV-4	Philippines<br>Vietnam	Pingtung County & Kaohsiung City<br>Pingtung County
**2005**	202	104	DENV-3<br>DENV-2<br>DENV-3	Philippines<br>Vietnam<br>Vietnam	Kaohsiung City<br>Tainan City<br>Kaohsiung City
**2006**	**965**	109	DENV-3<br>DENV-2	Cambodia<br>Vietnam	Kaohsiung City<br>Kaohsiung City
**2007**	**2,000**	179	DENV-1<br>DENV-2	Thailand<br>Vietnam	Tainan City<br>Tainan City
**2008**	488	226	DENV-1<br>DENV-1<br>DENV-2<br>DENV-1	Vietnam<br>Thailand<br>Cambodia<br>Vietnam	Kaohsiung City<br>Kaohsiung City<br>Kaohsiung City<br>Taipei City
**2009**	**848**	204	DENV-3<br>DENV-2<br>DENV-1	Philippines<br>Vietnam<br>Thailand	Southern Taiwan[b]<br>Pingtung County<br>Changhua County

**2010**	1,592	304	DENV-3<br>DENV-4<br>DENV-2<br>DENV-1<br>DENV-1	Philippines<br>Indonesia<br>Philippines<br>Vietnam<br>Cambodia	Kaohsiung City<br>Tainan City<br>Kaohsiung City<br>Tainan City<br>New Taipei City
**2011**	1,545	157	DENV-2<br>DENV-1<br>DENV-1<br>DENV-3	Vietnam<br>Central & South America<br>Myanmar<br>Malaysia	Pingtung County & Kaohsiung City<br>Tainan City & Kaohsiung City<br>Taipei City & New Taipei City<br>Pingtung County & Kaohsiung City
**2012**	1,271	207	DENV-1<br>DENV-2	Central and South America<br>Indonesia	Tainan City<br>Kaohsiung City
**2013**	596	264	DENV-2<br>DENV-1<br>DENV-2	Indonesia<br>Central and South America<br>Indonesia	Pingtung County, Kaohsiung & Tainan Cities<br>Pingtung County<br>Pingtung County
**2014**	**15,492**	240	DENV-1<br>DENV-2	Indonesia<br>Myanmar	Pingtung County, Kaohsiung & Tainan Cities<br>Kaohsiung City
**2015**	**43,419**	365	DENV-1<br>DENV-2	Indonesia<br>Indonesia	Kaohsiung City<br>Tainan City & Kaohsiung City

[a]Based on molecular epidemiology. [b]Southern Taiwan includes both Tainan City and Kaohsiung City.

[c]Only Sporadic cases or small clusters occurred in Taiwan, from 2016 to 2020 due to enhanced the integrated dengue surveillance

[d]1981 outbreak occurred in the isolated islet of Hsiao-Liu-Chiu of Pingtung County, because fishermen went to the Philippines and brought the DENV-2 virus back.
