# Supplementary material for: Use of seroprevalence to guide dengue vaccination plans for older adults in a dengue non-endemic country
Source: PLoS Negl Trop Dis. 2021 Apr 1;15(4):e0009312. doi: 10.1371/journal.pntd.0009312 (PMC8075253; doi:10.1371/journal.pntd.0009312)
Supplement: S2 Table — (PDF) [file pntd.0009312.s002.pdf]

S2 Table. Population sizes, population densities, dengue cases and district-specific incidence of dengue in Kaohsiung City and Tainan City, 2015.

Areas	Population Sizes (persons)	Population Densities (people per km^2)	Number of dengue cases in 2015	District-specific incidence in 2015 (per 1,000 population)
**Kaohsiung City**				
**Sanmin**	346,169	17495.12	4702	13.58
**Cianjhen**	192,593	10072.49	2725	14.15
**Lingya**	174,515	21407.10	2263	12.97
**Tainan City**				
**Annan**	191,196	1768.82	1845	9.73
**Anping**	65,813	5908.03	925	14.15
**East**	187,834	14078.01	3143	16.64
**Yongkang**	232,210	5730.71	2675	11.59
**South**	125,768	4611.18	3510	27.92
**North**	133,091	12713.34	5739	43.26
**West Central**	76,967	12379.87	3498	45.14

The data of population density and population size were obtained from open sources released by the Bureau of Civil Affairs, Tainan City Government and Kaohsiung City Government (https://bca.tainan.gov.tw/; https://cabu.kcg.gov.tw/ Web/StatRpts/ StatRpt1.aspx).
