# Supplementary material for: Use of seroprevalence to guide dengue vaccination plans for older adults in a dengue non-endemic country
Source: PLoS Negl Trop Dis. 2021 Apr 1;15(4):e0009312. doi: 10.1371/journal.pntd.0009312 (PMC8075253; doi:10.1371/journal.pntd.0009312)
Supplement: S3 Table — (PDF) [file pntd.0009312.s003.pdf]

S3 Table. Final results of DENV and/or JEV infection for the three samples, based on the ratio of OD values

Sample No.	OD Readings			DENV/CC	JEV/CC	Final Results Judged
	DENV	JEV	Cell Control			
**Z125**	0.8817	0.2392	0.2218	3.9752	1.0784	**DENV+/JEV-**
**Z132**	1.0402	0.4383	0.2012	5.16998	2.1784	**DENV+/JEV+**
**S383**	0.3657	1.1727	0.2887	1.26671	4.062	**DENV-/JEV+**

CC: Cell Control; **DENV:** Dengue Virus; **JEV:** Japanese Encephalitis Virus;

(+): Positive; **(-):** Negative

DENV OD value / mock-infected supernatants OD value ≥ 2: DENV (+)

DENV OD value / mock-infected supernatants OD value < 2: DENV (-)

JEV OD value / mock-infected supernatants OD value ≥ 2: JEV (+)

JEV OD value / mock-infected supernatants OD value < 2: JEV (-)
