# Supplementary material for: Use of seroprevalence to guide dengue vaccination plans for older adults in a dengue non-endemic country
Source: PLoS Negl Trop Dis. 2021 Apr 1;15(4):e0009312. doi: 10.1371/journal.pntd.0009312 (PMC8075253; doi:10.1371/journal.pntd.0009312)
Supplement: S4 Table — (PDF) [file pntd.0009312.s004.pdf]

S4 Table Five tested samples and their final results of DENV serotype or secondary DENV infection judged, based on the OD value ratio

ID	DENV-1	DENV-2	DENV-3	DENV-4	JEV	CC	OD Ratio	Final Results
E360	1.588	0.893	1.092	0.586	0.364	0.301	**1.4542**	DENV-1
N135	0.903	1.284	0.824	0.439	0.514	0.155	**1.4219**	DENV-2
Y735	0.921	0.728	1.237	0.879	0.25	0.135	**1.3431**	DENV-3
AP053	0.333	0.597	0.298	0.993	0.327	0.118	**1.6633**	DENV-4
AP158	1.368	1.591	1.576	0.504	0.553	0.403	1.0095	Secondary DENV
