# Supplementary material for: Use of seroprevalence to guide dengue vaccination plans for older adults in a dengue non-endemic country
Source: PLoS Negl Trop Dis. 2021 Apr 1;15(4):e0009312. doi: 10.1371/journal.pntd.0009312 (PMC8075253; doi:10.1371/journal.pntd.0009312)
Supplement: S5 Table — (PDF) [file pntd.0009312.s005.pdf]

S5 Table. Calculation process and steps to obtain the age-standardized DENV-IgG seroprevalence in Kaohsiung City and Tainan City, 2016.

Tainan (TN) in 2016						
**Age (years)**	**No tested**	**No of DENV-Ab(+)**	**Crude rate**	**Std. Pop. in TN & KH**	**Expected Ab (+)**	**Age-standardized DENV seroprevalence**
40-49	387	28	7.23514212	728174	52684.42377	7.235142119
50-59	776	47	6.05670103	734376	44478.95876	6.056701031
60-69	1000	95	9.5	539731	51274.445	9.5
70-79	402	103	25.6218905	251906	64543.0796	25.62189055
80-89	38	18	47.3684211	122512	58032	47.36842105
**Total**	**2603**	**291**	**11.1794084**	**2376699**	**271012.9071**	**11.40291249**

Kaohsiung (KH) in 2016						
**Age (years)**	**No tested**	**No of DENV-Ab(+)**	**Crude rate**	**Std. Pop. in TN & KH**	**Expected Ab (+)**	**Age-standardized DENV seroprevalence**
40-49	34	5	14.7058824	728174	107084.4118	14.70588235
50-59	98	23	23.4693878	734376	172353.551	23.46938776
60-69	503	130	25.8449304	539731	139493.1014	25.84493042
70-79	645	318	49.3023256	251906	124195.5163	49.30232558
80-89	202	116	57.4257426	122512	70353.42574	57.42574257
90-99*	16	3	18.75	10220	1916.25	18.75
**Total**	**1498**	**595**	**39.7196262**	**2386919**	**615396.2562**	**25.7820335**

* Age group 90-99 years was calculated only in Kaohsiung, because there was no sample in this age group in Tainan.

[a]**Data sources:** Department of Household Registration, Ministry of the Interior, Taiwan. <https://www .ris.gov.tw/app/portal/346>

The Four Steps in Calculation Process:

Step 1: Searched for the population data of Kaohsiung City (KH) and Tainan City (TN) in the mid-year of 2016 (i.e. at the end of June, 2016) from the websites of Department of Household Registration, Ministry of the Interior, Taiwan**. Added up the age-specific population data of the two cities serving as the standard population (std. pop.)**

Step 2: Obtained DENV Seroprevalence (%) before standardization from Table 2.

Step 3: Used this DENV Seroprevalence (%) (from step 2) multiplied by Standard Population (from step 1) resulted in the **age-specific expected numbers of persons with of DENV Ab (+).**

Step 4: Added up all the expected numbers of persons with DENV Ab (+) (from step 3) divided by the total no of the Standard Population (from step 1) and thus obtained the age-standardized DENV Seroprevalence in the two cities.
