# Supplementary figures and images for: Use of seroprevalence to guide dengue vaccination plans for older adults in a dengue non-endemic country
Source: PLoS Negl Trop Dis. 2021 Apr 1;15(4):e0009312. doi: 10.1371/journal.pntd.0009312 (PMC8075253; doi:10.1371/journal.pntd.0009312)

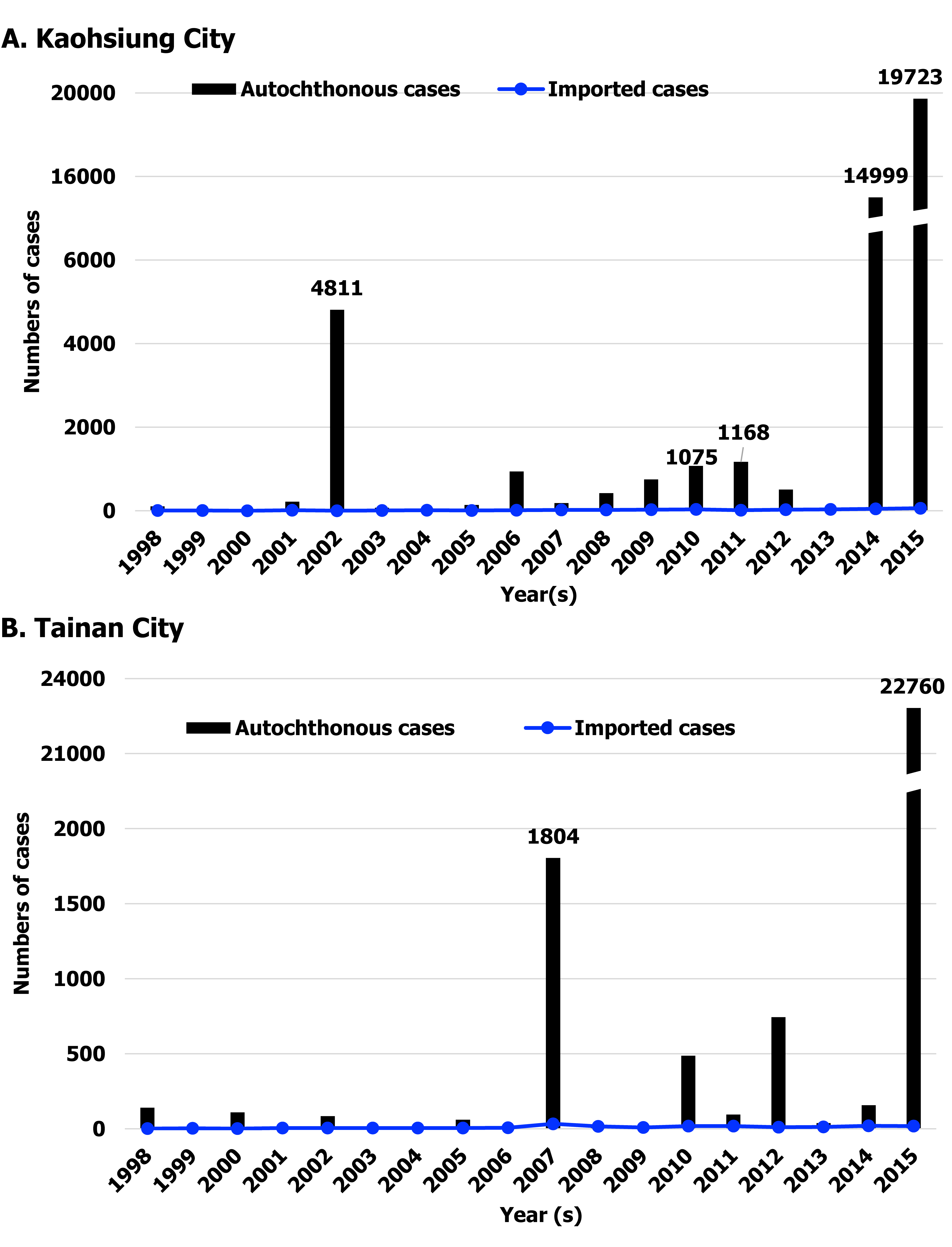

Supplement: S1 Fig — (TIF) [file pntd.0009312.s007.tif]

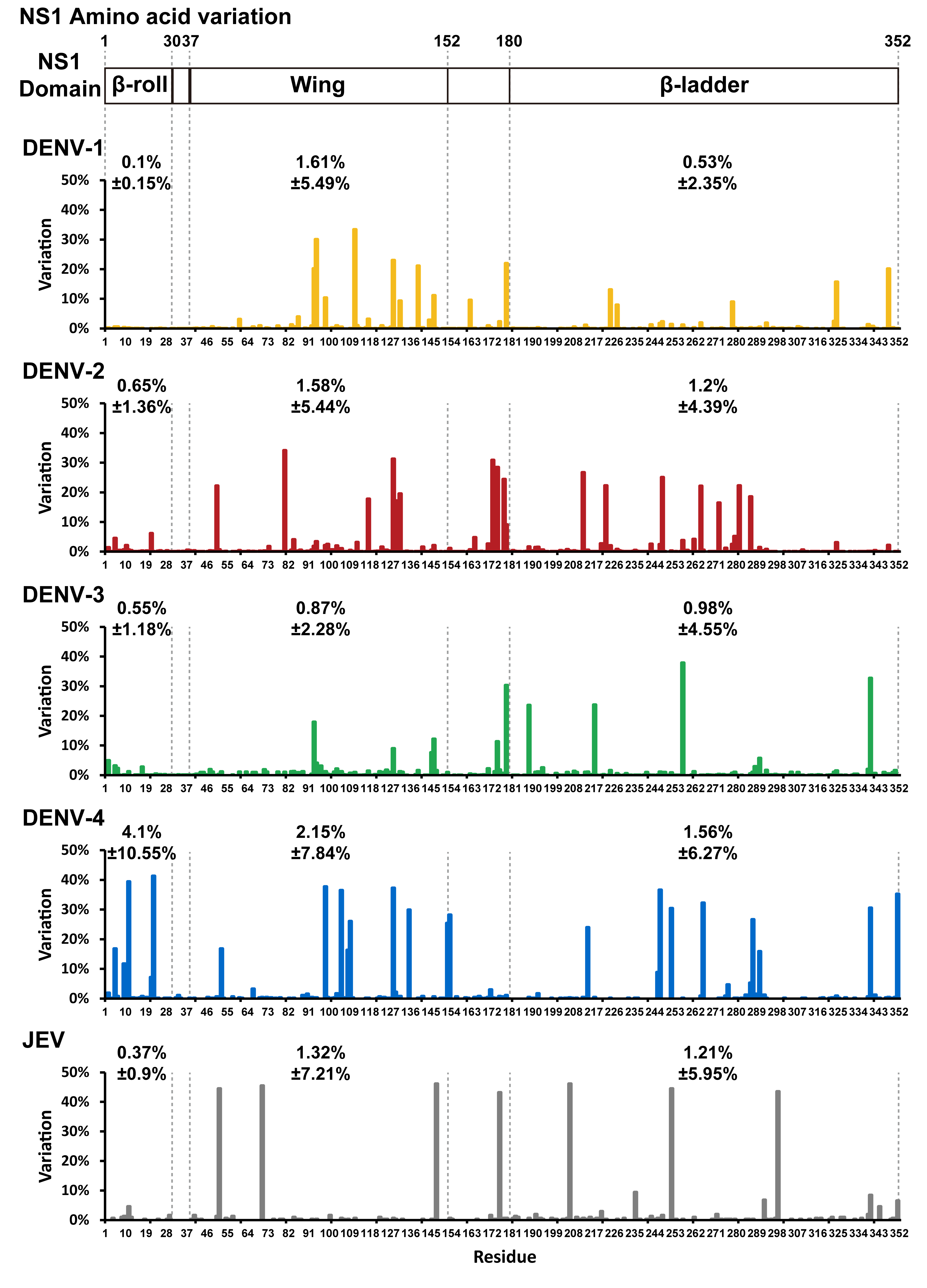

Supplement: S2 Fig — In total, 9,762 DENV strains and 308 JEV strains collected worldwide from the ViPR database (during 1950–2020) [88] with known DENV serotype and available NS1 sequences were analyzed. These included 4,085 DENV-1 strains (12 Taiwan strains), 3,259 DENV-2 strains (107 Taiwan strains), 1,776 DENV-3 strains (17 Taiwan strains), 642 DENV-4 strains (2 Taiwan strains), and 308 JEV strains (30 Taiwan strains). The three domains of NS1 are: (1) β-roll domain [amino acid (a.a.) residues 1–30], (2) Wing domain [a.a. residues 37–152], and (3) β-ladder domain [a.a. residues 180–352]. The plot shows that the Wing domain of DENV NS1 protein had the highest a.a. variation. Among the four DENV serotypes, DENV-4 had the highest a.a. variation percentages in all the three domains of the NS1 protein. In addition, both DENV-1 and DENV-2 had the highest a.a. variations in the Wing domain, whereas DENV-3 showed the greatest a.a. variation in the β-ladder domain; and DENV-4 revealed the widest a.a. variation in the β-roll domain. The values of mean ± standard deviation (mean ± SD) are shown for each of the three domains for all of four of the DENV serotypes. The methods used to calculate the percentage of amino acid variations were described previously [87]. (TIF) [file pntd.0009312.s008.tif]

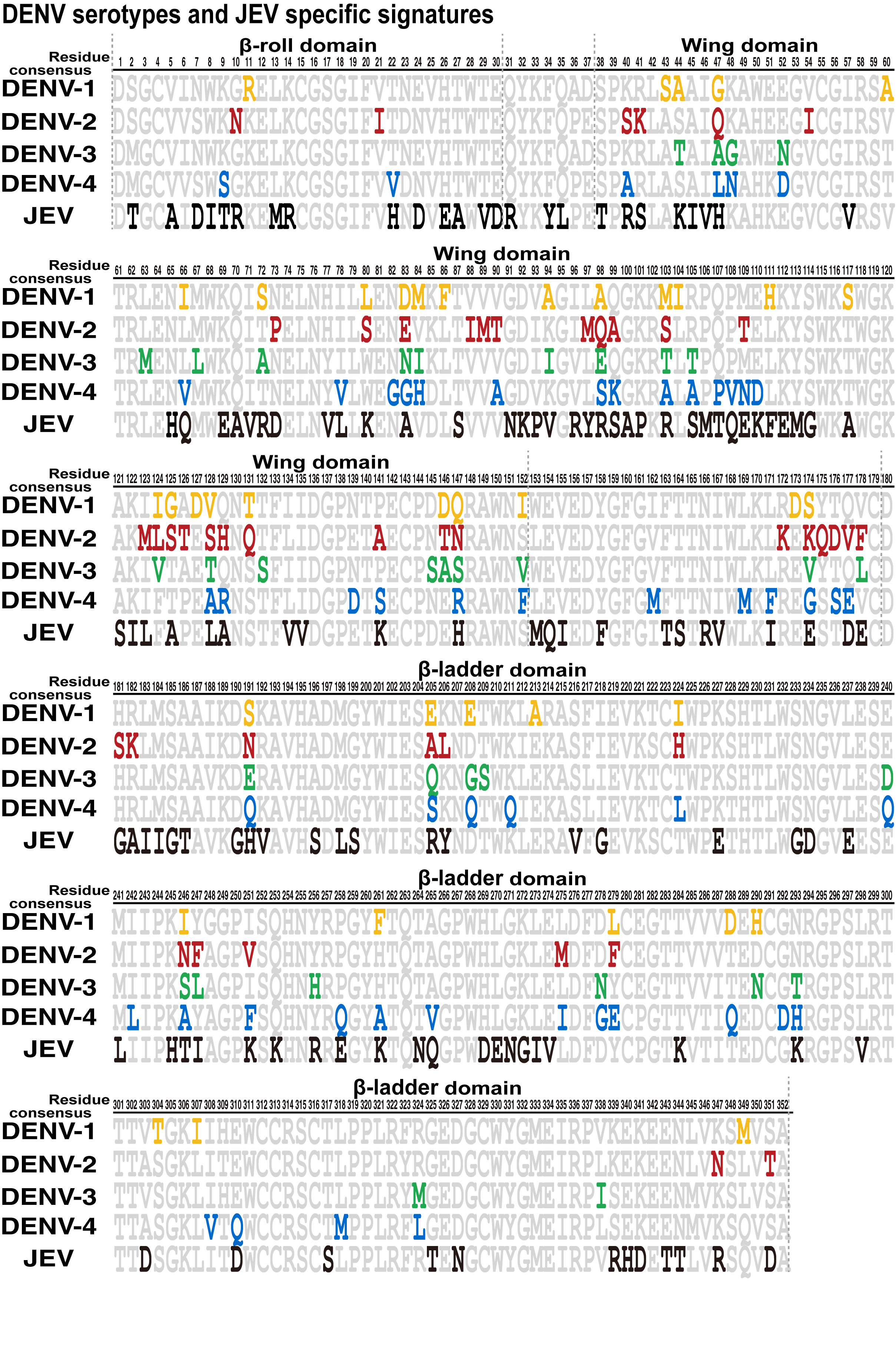

Supplement: S3 Fig — The DENV serotype- and JEV-specific consensus amino acid signatures for each group are shown by the constellation of the 352 amino acid residues in NS1 protein (distinguished by darkened colors). The plot shows that the Wing domain had the highest signatures to differentiate the DENV serotypes from JEV. Conversely, β-ladder domain sequence was more conserved among the four DENV serotypes. The methods used to find signatures were described previously [87]. (TIF) [file pntd.0009312.s009.tif]

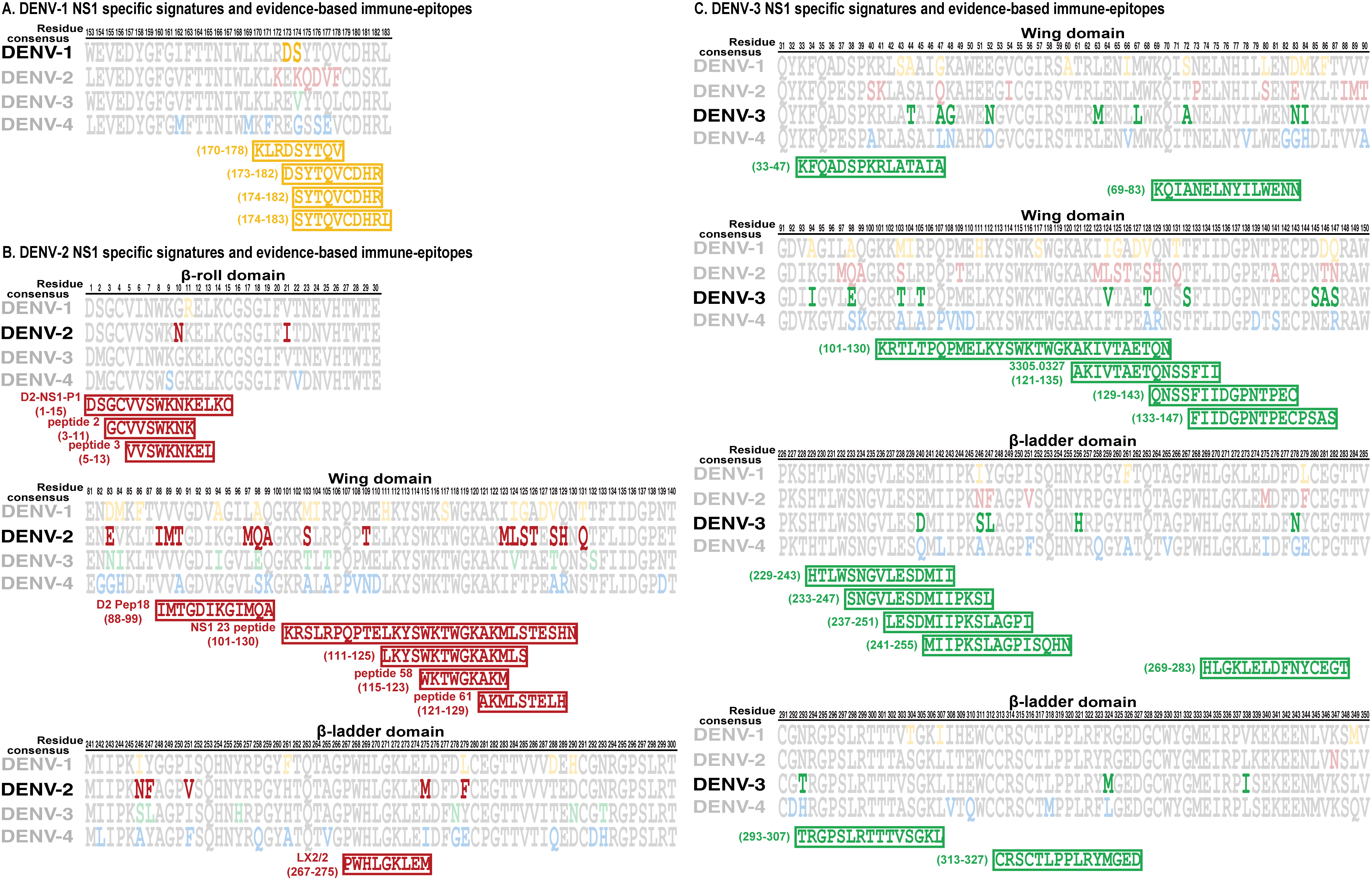

Supplement: S4 Fig — The DENV-1-, DENV-2-(Fig. S4A), and DENV-3 (Fig S4B)-specific consensus amino acid signatures for each DENV serotype are shown by the constellation of the 26 evidence-based immune-epitopes documented in the literature, which are listed in the letters within the boxes. All evidence supporting information from the raw experimental data for these immunological epitopes is listed in Supporting Information file from ViPR databases [88]. (TIF) [file pntd.0009312.s010.tif]
