# Supplementary material for: Use of seroprevalence to guide dengue vaccination plans for older adults in a dengue non-endemic country
Source: PLoS Negl Trop Dis. 2021 Apr 1;15(4):e0009312. doi: 10.1371/journal.pntd.0009312 (PMC8075253; doi:10.1371/journal.pntd.0009312)
Supplement: S1 Protocol — (PDF) [file pntd.0009312.s011.pdf]

The detail laboratory procedures of the three serological assays used in this study are described as follows.

(1) Dengue Virus IgG indirect enzyme-linked immunosorbent assay (ELISA)

A total of 1498 samples from Kaohsiung City were first tested for DENV-specific IgG antibodies by using a commercial indirect ELISA (Focus Dengue Virus IgG DxSelect, USA), according to the manufacturer’s instructions. The sensitivity and specificity of this test were 96% and 93%, respectively [1].

(2) DENV/JEV NS1-specific indirect IgG ELISA

To understand possible cross-reactivity between DENV and Japanese encephalitis virus (JEV), we further tested the anti-DENV-IgG-positive samples from Focus Dengue Virus IgG DxSelect by a NS1-indirect IgG ELISA that can differentiate between DENV and JEV in accordance with following the protocols as listed in the previous study [2]. We also used the monoclonal antibody (mAb) D2/8-1 (bought from YH0023 against NS1 protein of DENV [3], Yao-Hong Biotechnology Inc.) as the NS1-capture antibody for testing the anti-DENV-IgG-positive samples from Kaohsiung and all the 2603 samples collected directly from Tainan. In brief, each well was coated with 5 μg/ml, 100 μl/well of mAb D2/8-1, which recognizes a conserved linear epitope of NS1 protein among the four DENV serotypes and JEV [4-6] in 0.1M carbonate (Na_2CO_3-$NaHCO_3$, pH = 9.5) overnight at 4°C. After washing with phosphate buffered saline with 0.05% Tween™ 20 (PBST), the wells were blocked with 200 μl of PBS-

1% bovine serum albumin (BSA) for 1 h at 37 °C. After washing, the wells were incubated with 100 µl 1x10^5 plaque forming unit (PFU)/well NS1-containing culture supernatant mixture of the four Taiwan DENV local strains of DENV-1 (8700828), DENV-2 (454009), DENV-3 (8700829), and DENV-4 (8700544), or JEV (JaGar)-infected C6/36 cells for 30 minutes at 37 °C. After washing, 1:50 diluted human serum samples were added and incubated for 30 minutes at 37 °C, followed by washing and adding 100µl/well goat anti-human IgG-horseradish peroxidase (HRP) (1:20000, Jackson ImmunoResearch Labortories, West Grove, PA, USA) and incubated for 30 min at 37 °C. The color was developed by adding 3, 3′, 5, 5′-Tetramethylbenzidine (TMB) substrate and 3N HCl as a stop reagent. The absorbance was read at the wavelength of 450 nm. DENV-seropositive and DENV-seronegative results were obtained, according to the criteria of the Centers for Disease Control in Taiwan (Taiwan-CDC) [7]. In addition, the seroprevalence of DENV-IgG from 2603 Tainan serum samples was measured directly using the aforementioned DENV/JEV NS1-specific indirect IgG ELISA test, as described above for cost consideration (2nd and 3rd assays in the Figure 2). The solid examples of data analyses are described in the **S3 Table**.

(3) DENV-NS1 serotype-specific IgG ELISA test.

To further investigate the exposed DENV serotypes and secondary DENV infection simultaneously among older adults, we selected 188 and 105 serum samples with positive DENV-NS1-IgG from Kaohsiung and Tainan, respectively considering variations in age

groups and residential districts, to conduct a DENV NS1 serotype-specific IgG ELISA, based on the protocols of the Taiwan-CDC [7]. Briefly, each well was coated with 5 μg/ml, 100 μl/well of mAb D2/8-1 in 0.1M carbonate (Na_2CO_3-$NaHCO_3$, pH = 9.5) overnight at 4 °C [8] After washing with PBST for 4 times, the wells were blocked with 200 μl of phosphate-buffered saline-1% bovine serum albumin (BSA) for 1 hour at 37 °C. After washing, the wells were added with 1:3 diluted NS1-containing culture supernatants of Taiwan local dengue strains of DENV-1 (8700828), DENV-2 (454009), DENV-3 (8700829), DENV-4 (8700544) infected Vero cells into the four different wells for 30 minutes at 37 °C. After washing with PBST for four times, 1:100 diluted serum samples were added and incubated for 30 minutes at 37 °C, followed by adding 100 μl/well goat anti-human IgG-alkaline phosphatase (1:5000, Jackson ImmunoResearch Laboratories, West Grove, PA, USA) and incubated for 30 min at 37°C. The color was developed by adding substrate p-nitrophenylphosphate (100 μl/well; Chemicon, Cat.ES009), and the optical density was determined within 40 minutes at the wavelength of 405 nm and 620 nm.

The performance of this assay has been shown to be correlated well with dengue virus plaque reduction neutralization test (PRNT) [89.7% (73+32/117) consistency of primary and secondary DENV infection, and the correlation of Kappa statistics = 0.776] [9]. Examples of the data from this DENV-NS1-based serotyping assay are provided (S4 Table) to elucidate the algorithm in determination of DENV serotype and secondary DENV infection.

S1 Protocol References

1. Focus Diagnostics C, CA, USA. Dengue Virus Serology Testing. Available from: https://www.focusdx.com/pdfs/brochures/DXDENI0511_Dengue_Virus_Serology.pdf.
2. Bureau of Civil Affairs KCG. Available from: https://cabueng.kcg.gov.tw/.
3. Chen LK, Liao CL, Lin CG, Lai SC, Liu CI, Ma SH, et al. Persistence of Japanese encephalitis virus is associated with abnormal expression of the nonstructural protein NS1 in host cells. Virology. 1996;217(1):220-9. Epub 1996/03/01. doi: 10.1006/viro.1996.0109. PubMed PMID: 8599206.
4. Chen WJ. Dengue outbreaks and the geographic distribution of dengue vectors in Taiwan: A 20-year epidemiological analysis. Biomed J. 2018;41(5):283-9. Epub 2018/12/26. doi: 10.1016/j.bj.2018.06.002. PubMed PMID: 30580791; PubMed Central PMCID: PMCPMC6306330.
5. Shu PY, Chien LJ, Chang SF, Su CL, Kuo YC, Liao TL, et al. Fever screening at airports and imported dengue. Emerg Infect Dis. 2005;11(3):460-2. Epub 2005/03/11. doi: 10.3201/eid1103.040420. PubMed PMID: 15757566; PubMed Central PMCID: PMCPMC3298237.
6. Shang CS, Fang CT, Liu CM, Wen TH, Tsai KH, King CC. The role of imported cases and favorable meteorological conditions in the onset of dengue epidemics. PLoS Negl Trop Dis. 2010;4(8):e775. Epub 2010/08/07. doi: 10.1371/journal.pntd.0000775. PubMed PMID: 20689820; PubMed Central PMCID: PMCPMC2914757.
7. Shu PY, Chen LK, Chang SF, Yueh YY, Chow L, Chien LJ, et al. Dengue NS1-specific antibody responses: isotype distribution and serotyping in patients with Dengue fever and Dengue hemorrhagic fever. J Med Virol. 2000;62(2):224-32. Epub 2000/09/26. doi: 10.1002/1096-9071(200010)62:2<224::aid-jmv14>3.0.co;2-c. PubMed PMID: 11002252.
8. Yen TY, Trovoada dos Santos Mde J, Tseng LF, Chang SF, Cheng CF, Carvalho AV, et al. Seroprevalence of antibodies against dengue virus among pregnant women in the Democratic Republic of Sao Tome and Principe. Acta Trop. 2016;155:58-62. Epub 2016/01/08. doi: 10.1016/j.actatropica.2015.12.012. PubMed PMID: 26739653.
9. Shu PY, Chen LK, Chang SF, Yueh YY, Chow L, Chien LJ, et al. Potential application of nonstructural protein NS1 serotype-specific immunoglobulin G enzyme-linked immunosorbent assay in the seroepidemiologic study of dengue virus infection: correlation of results with those of the plaque reduction neutralization test. J Clin Microbiol. 2002;40(5):1840-4. Epub 2002/05/01. doi: 10.1128/jcm.40.5.1840-1844.2002. PubMed PMID: 11980973; PubMed Central PMCID: PMCPMC130672.
