# Supplementary material for: Use of seroprevalence to guide dengue vaccination plans for older adults in a dengue non-endemic country
Source: PLoS Negl Trop Dis. 2021 Apr 1;15(4):e0009312. doi: 10.1371/journal.pntd.0009312 (PMC8075253; doi:10.1371/journal.pntd.0009312)
Supplement: S6 Table — (PDF) [file pntd.0009312.s006.pdf]

S6 Table. Geographical variations in DENV serotypes and secondary DENV infection in the five age groups in Kaohsiung and Tainan cities of southern Taiwan, 2016.

Years of Birth	Age Groups	Kaohsiung City						Tainan City					
		**DENV-1**	**DENV-2**	**DENV-3**	**DENV-4**	**2ndary [a]**	**Total**	**DENV-1**	**DENV-2**	**DENV-3**	**DENV-4**	**2ndary[a]**	**Total**
**After 1951**	**<65**	3	1	0	0	5	9	1	17	0	1	7	26
**1947-1951**	**65-69**	9	10	1	0	15	35	0	17	0	1	11	29
**1942-1946**	**70-74**	12	10	6	0	22	50	2	7	1	0	11	21
**1937-1941**	**75-79**	18	6	7	0	21	52	1	0	3	0	16	20
**Before 1936**	**≥80**	13	7	4	0	18	42	3	1	3	0	2	9
**Total (%)**		55 (29.3)	34 (18.1)	18 (9.6)	0 (0)	81 (43.09)	188	7 (6.67)	42 (40)	7 (6.67)	2 (1.90)	47 (44.76)	105

[a] **“2ndary” DENV secondary infection** means more than one DENV infection without clearly knowing DENV serotypes.
